# Mitophagy-Mediated Tumor Dormancy Protects Cancer Cells from Chemotherapy

**DOI:** 10.3390/biomedicines12020305

**Published:** 2024-01-28

**Authors:** Yunqing Sun, Yang Chen, Zhenan Liu, Jingjing Wang, Junqiang Bai, Ruixue Du, Mingshu Long, Zhengjun Shang

**Affiliations:** 1 State Key Laboratory of Oral & Maxillofacial Reconstruction and Regeneration, Key Laboratory of Oral Biomedicine Ministry of Education, Hubei Key Laboratory of Stomatology, School & Hospital of Stomatology, Wuhan University, Wuhan 430079, China; yunqingsun@whu.edu.cn (Y.S.); chenyang1994@whu.edu.cn (Y.C.); 2021103040021@whu.edu.cn (Z.L.); 2021283040100@whu.edu.cn (J.W.); junqiangbai@whu.edu.cn (J.B.); drxwz320@163.com (R.D.); lmsdent@163.com (M.L.); 2 Department of Oral and Maxillofacial Surgery, School & Hospital of Stomatology, Wuhan University, Wuhan 430079, China; 3 Department of Oral and Maxillofacial-Head and Neck Oncology, School & Hospital of Stomatology, Wuhan University, Wuhan 430079, China

**Keywords:** mitophagy, dormancy, chemotherapy, HNSCC

## Abstract

Despite obvious tumor shrinkage, relapse after chemotherapy remains a main cause of cancer-related mortality, indicating that a subpopulation of cancer cells acquires chemoresistance and lingers after treatment. However, the mechanism involved in the emergence of chemoresistant cells remains largely unknown. Here, we demonstrate that the degradation of mitochondria via autophagy leads to a dormant state in a subpopulation of cancer cells and confers on them resistance to lethal cisplatin (DDP) exposure. The surviving DDP-resistant cells (hereafter, DRCs) have a lower metabolic rate but a stronger potential malignant potential. In the absence of DDP, these DRCs exhibit an ever-increasing self-renewal ability and heightened tumorigenicity. The combination of chloroquine and DDP exerts potent tumor-suppressive effects. In summary, our findings illuminate the mechanism between mitophagy and tumor dormancy and prove that targeting mitophagy might be a promising approach for overcoming chemoresistance in head and neck squamous cell carcinoma (HNSCC).

## 1. Introduction

Cisplatin (DDP) is a frontline therapeutic drug for treating head and neck squamous cell carcinoma (HNSCC) [1]. It exerts its anti-tumor effects through DNA damage response and mitochondria-induced apoptosis in tumor cells [2]. However, despite the initial success of DDP chemotherapy, tumor recurrence and further metastasis remain the primary reasons for treatment failure and patient mortality [3]. This process involves a small subpopulation of tumor cells evading surgery and chemotherapy, resettling either in situ or within the circulatory system. The recurrence of tumors post-treatment reflects a tolerance to the existing therapeutic approaches, linked to the selection of drug-resistant cells, as well as the metabolic heterogeneity and high plasticity of tumor cells [4]. The mechanisms behind tumor resistance to chemotherapy and the subsequent resurgence of tumors may be linked to the emergence of drug-resistant cells [5]. Our previous research has demonstrated the protective role of autophagy in tumor cells under various harsh conditions, including DDP treatment, with an observed increase in autophagy levels in DDP-treated cells [6]. Following extensive metastasis, surgical and radiation treatments become impractical, and resistance to drugs entering the circulatory system develops. Further discussion is needed to explore the relationship between autophagy and DDP-resistant cells.

Mitophagy, also known as mitochondrial autophagy, is the process of autophagy that occurs specifically in mitochondria [7]. Its purpose is to degrade damaged mitochondria and regulate mitochondrial quantity, playing a crucial role in maintaining mitochondrial and cellular homeostasis [8]. Mitochondria are the central sites for oxidative phosphorylation, generating reactive oxygen species as byproducts, which can lead to mitochondrial damage and dysfunction [9]. The dynamic equilibrium between mitophagy and mitochondrial biogenesis plays a crucial role in maintaining the normal functioning of physiological activities [10]. The PTEN-induced putative kinase 1 (PINK1)/Parkin pathway is currently the most extensively studied ubiquitin-dependent pathway in the mitophagy process [11]. The *PARK2* gene encodes the E3 ubiquitin ligase Parkin protein, which mediates ubiquitination and degradation by connecting ubiquitin molecules to substrates. Meanwhile, PINK1 accumulates on the outer mitochondrial membrane and catalyzes Parkin activation in response to mitochondrial membrane potential damage, promoting ubiquitination and degradation of mitochondria. In addition to the ubiquitination pathway, non-ubiquitin-dependent autophagic pathways involving proteins like NIX, BNIP3, and FUNDC1 also participate in mitochondrial autophagy [8,12,13,14]. Mitochondrial autophagy has been found to be associated with various diseases, including neurodegenerative diseases, cardiovascular diseases, metabolic diseases, innate immunity, etc. [8,15,16,17]. Additionally, mitophagy plays a crucial role in cancer by eliminating damaged mitochondrial DNA, alleviating oxidative stress, and reducing carcinogenesis [18]. On the other hand, mitophagy can provide a protective mechanism for tumor cells, aiding in their survival under adverse external conditions [19]. We have observed an elevation in mitophagy during the DDP treatment process, suggesting a potential association with the enhancement of tumor drug resistance.

Dormant cells are a subset of cells within tumors that exhibit slow or halted proliferation. They are characterized by residing in the G0/G1 phase of the cell cycle and an increase in cell cycle protein-dependent kinase inhibitors, such as p21 [20,21]. Dormant cells play a crucial role in tumor resistance and residual microlesions [22]. They exhibit a strong correlation with tumor recurrence and distant metastasis [4]. The theory of dormant cells shares similarities with the concept of cancer stem cells (CSCs). CSCs have the ability to self-renew and differentiate, which contributes to tumor resistance, survival, and the acquisition of enhanced proliferative and invasive capabilities. Within the CSC population, there is a subset that exhibits quiescent capabilities, showing increased tolerance to the environment and the ability to transition between dormancy and proliferation states [23]. Dormant states are observed throughout various stages of tumor initiation and progression [24]. In the primary tumor site, cancer cells alternate between a quiescent and proliferative state, facilitating better adaptation to the microenvironment and the accumulation of mutations. Some tumor cells enter the circulatory system, transitioning into a quiescent state in the bloodstream or distant microenvironments. In the context of tumor drug therapy, treatment-induced dormancy aids certain tumor cells in evading the effects of drugs and surviving, resulting in the suppression of a substantial number of proliferating cells but failing to prevent tumor recurrence [25]. We have observed that there is a collective transition into a dormant state in DDP-treated tumor cells, enhancing the resistance and survival capabilities of tumor cells.

In this study, we identified an elevated level of mitochondrial autophagy in DDP-treated HNSCC cell lines. The increased mitochondrial autophagy played a role in causing the tumor cells to enter a dormant state, which in turn led to heightened resistance and enhanced CSC potential. Furthermore, upon the conclusion of the dormant state, the tumor cells exhibited a higher malignancy and increased tumorigenicity.

## 2. Materials and Methods 

### 2.1. Cell Lines and Culture

The CAL27 and FaDu cell lines were purchased from the ATCC (American Type Culture Collection, Rockville, MD, USA). CAL27 cells were cultured in a high-glucose DMEM medium supplemented with 10% fetal bovine serum, and FaDu cells were cultured in FaDu-specific medium (CM-0083, Pricella, Wuhan, China). The cultures were maintained at 37 °C in a 5% CO_2_ atmosphere. To induce a certain level of drug resistance, both cell lines were treated with low concentrations (1/10 of the IC50) of cisplatin (DDP) for five consecutive passages before subsequent experiments.

### 2.2. IC50 Value Assay

The cells were seeded onto a 96-well plate, and after cell adherence, the culture medium and cisplatin were replaced. After 48 h, the CCK-8 kit (BS350B, Biosharp, Hefei, China) was employed to measure and calculate the results. This was repeated three times for each set (*n* = 3). Statistical analysis of the data was performed using GraphPad 9.0. The CCK-8 assay was conducted according to the instructions provided in the protocol.

### 2.3. Cell Fluorescence

The cells were seeded onto confocal dishes, and after adherence, they were cultured with or without cisplatin (at a specified concentration) for 24 h. Subsequently, mitochondrial and lysosomal staining was performed using MitoTracker Green (C1048, Beyotime, Shanghai, China) and LysoTracker Red (C1046, Beyotime). These dyes were individually diluted in HBSS with Ca^2+^ and Mg^2+^ (C2019, Beyotime) to concentrations of 1:25,000 and 1:15,000, respectively. After incubation at 37 °C for 40 min, the medium was replaced with Hoechst (C1011, Beyotime) diluted at 1:1000, followed by an 8 min incubation. The cells were then washed three times with PBS and subjected to confocal microscopy for analysis.

### 2.4. Glucose Consumption and Lactate Production Assay

Cells from the negative control group and cisplatin-resistant cells post-treatment were seeded onto a 6-well plate. After adherence, the cells were cultured for 48 h. The supernatant was collected, and glucose content was measured using the Glucose Content Detection Kit (BC2500, Solarbio, Beijing, China), while lactate content was determined using the Lactate Content Detection Kit (A019-2-1, Jiancheng Bio, Nanjing, China). This was repeated three times for each set (*n* = 3). The detailed procedures were followed as per the instructions provided with the respective assay kits.

### 2.5. Western Blot

We conducted experiments following previously published procedures [26]. The antibodies pRB (30376-1-AP), CyclinE1 (11554-1-AP), and p21 (10355-1-AP) were procured from Proteintech (Rosemont, IL, USA).

### 2.6. EdU Assay

Cells were seeded on a 12-well plate and cultured for 48 h. Afterward, EdU 2X working solution, prepared in the same volume as the culture medium, was added. The cells were then incubated at 37 °C for 2 h and fixed by 4% paraformaldehyde for 15 min. After washing them 3 times, the cells were subjected to Click Reaction Solution at room temperature in the dark for 30 min, followed by another 3 washes. Hoechst (1:1000) was used for an 8 min incubation. This was repeated three times for each set (*n* = 3). Detailed procedures were conducted following the guidance provided in the EdU Kit (C0078, Beyotime).

### 2.7. Cell Cycle Assay

After culturing, the cells were digested with trypsin and fixed in 1 mL of 70% ethanol at 4 °C for 2 h. Following centrifugation at 1000× *g* for 3 min, the supernatant was removed, and 1 mL of pre-chilled PBS was added. Propidium staining solution (C1052, Beyotime) was prepared, and 0.5 mL of the staining solution was added to each sample, followed by incubation at 37 °C in the dark for 30 min. The samples were placed on ice and then flow cytometry analysis was performed. This was repeated three times for each set (*n* = 3).

### 2.8. Colony Formation Assay

Cells were seeded onto a 12-well plate and cultured in a 10% fetal bovine serum medium for 10 days. Representative images were observed and captured under a microscope. The cells were fixed with 4% paraformaldehyde for 20 min, followed by three washes with PBS. Crystal violet staining was performed for 10 min, and the plate was then washed and air-dried. This was repeated three times for each set (*n* = 3). Cell images were captured, and colony area was quantified using Image J.

### 2.9. In Vitro Limiting Dilution Assay

We pre-treated a 96-well plate with polyHEMA (10 g/L in 95% ethanol; MilliporeSigma, Darmstadt, Germany), adding 100 μL to each well. Cells were cultured in standard CSC medium [26], and after 7 d, the number of spheres was quantified. The specific procedures followed the Bioprotocol [27].

### 2.10. Mouse Xenografts

The animal experiment was approved by the Ethics Committee of the Hospital of Stomatology at Wuhan University (approval code: S07922090L). 

For the in vivo limiting dilution assay, 24 *BALB/c nude mice* (purchased from Vital River, China, Beijing, China) were employed. NC and DPRs (CAL27), at cell quantities of 10^3^, 10^4^, and 10^5^, were injected subcutaneously on the left or right side of the mice with 50 μL PBS and 50 μL matrigel. Tumors were harvested 35 d after tumor cell injection.

For the in vivo assay to test the role of autophagy in DDP resistance, another 18 *BALB/c nude mice* were used and divided into NC, DDP (cisplatin), and DDP+CQ (cisplatin plus chloroquine) groups (*n* = 6) to investigate the relationship between autophagy and DDP; each mouse was subcutaneously injected with 100 mL PBS containing 2 × 10^6^ CAL27 cells. Seven days after tumor formation, the DDP and DDP+CQ groups received intraperitoneal injections, every 3 days for 3 weeks, with doses of 4 mg/kg cisplatin and 4 mg/kg cisplatin + 40 mg/kg chloroquine. The NC group received PBS. Tumors were harvested 35 d after tumor cell injection.

### 2.11. Statistical Analysis

Experimental data of the limiting dilution assay were processed using Extreme Limiting Dilution Analysis software [28]. Available from: http://bioinf.wehi.edu.au/software/elda/index.html (accessed on 21 January 2024). Other statistics were analyzed and plotted using Graphpad 9.0. The Student’s t-test was used to analyze data between two groups. One-way analysis of variance was used to analyze data between multiple groups. All results are presented as means ± SEMs. Differences with *p*-values < 0.05 were considered statistically significant (* *p* < 0.05, ** *p* < 0.01, and *** *p* < 0.001).

## 3. Results

### 3.1. Mitophagy Contributes to the Formation of DDP-Resistant Cells

**Figure 1:**
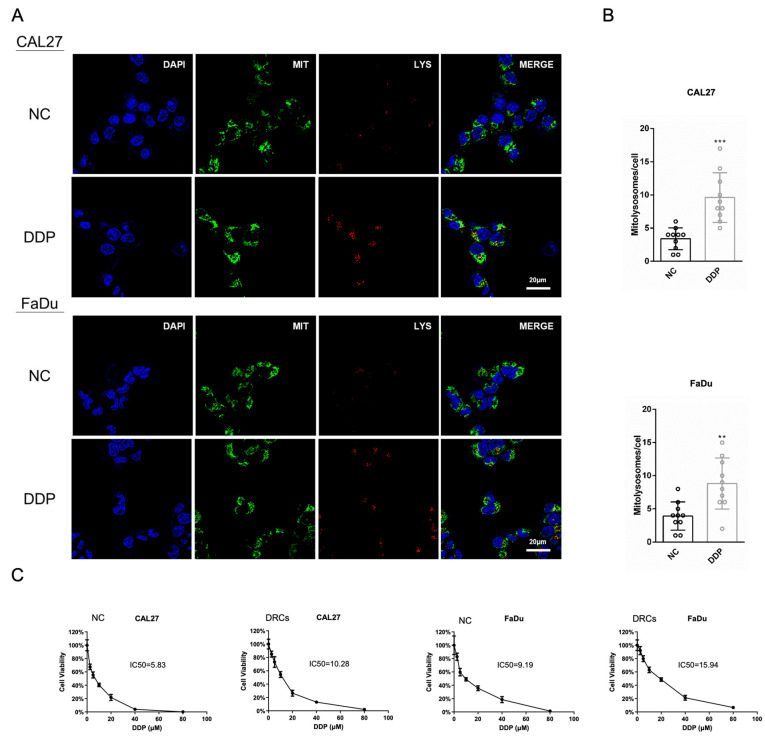
(**A**,**B**) Representative images of mitochondrial (MIT) and lysosomal (LYS) staining in the NC (Negative Control) and DDP (Cisplatin)-treated groups (**A**) along with the quantitative analysis of mitolysosome counts (**B**) of CAL27 and FaDu cells. (**C**) The IC50 determination values of DDP in the DRC (DDP-resistant cell) and NC groups of CAL27 and FaDu cells. *n* = 3. ** *p* < 0.01 and *** *p* < 0.001.

During physiological or pathological processes, the autophagic system targets mitochondria, leading to their degradation in lysosomes. This process is known as mitochondrial autophagy or mitophagy [29]. An increase in autophagic flux was observed during the DDP treatment of HNSCC cell lines CAL27 and FaDu, using Mito-Tracker (Green) and Lyso-Tracker (Red) to trace mitochondria and lysosomes, respectively. On representative photomicrographs, one can see that the lysosomes (red) in the DDP group show a significant increase, indicating an increased level of autophagy. In addition, the lysosomes are highly co-localized with mitochondria (green), suggesting that this autophagy primarily occurs within the mitochondria. The study revealed a significant correlation between the occurrence of autophagy and the location of mitochondria during DDP treatment (Figure 1A,B), indicating an upregulation of mitophagy in tumor cells. When cancer cells encounter microenvironmental stress, they undergo metabolic and genetic changes to evade death, making complete tumor eradication difficult [30]. We subjected CAL27 and FaDu cells to prolonged exposure to low concentrations of DDP, and our observations revealed a significant increase in the IC50 of DDP (Figure 1C). These cells, tentatively named DDP-resistant cells (DRCs), were used in subsequent experiments. Elevated levels of mitophagy in DDP-treated HNSCC are associated with mitochondrial damage and metabolic remodeling. The findings suggest that mitophagy not only eliminates metabolically damaged mitochondria but also reduces the metabolic activity of tumor cells, inducing a state of low metabolism and reduced substance exchange similar to dormancy.

### 3.2. DRCs Exhibit Characteristics of Dormancy

**Figure 2:**
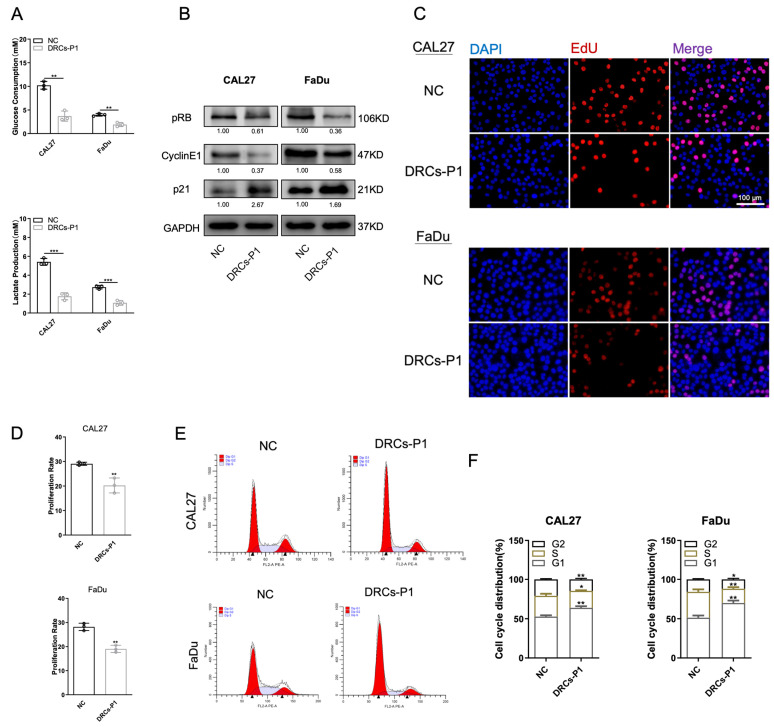
(**A**) Glucose consumption and lactate production in the NC (Negative Control) and DRC (DDP-resistant cell) groups of CAL27 and FaDu cells. *n* = 3. (**B**) Western blot results for pRB, CyclinE1, and p21 in NC and DRC groups of CAL27 and FaDu cells. (**C**,**D**) Representative images (**C**) and quantitative analysis (**D**) of Edu experiments in NC and DRC groups of CAL27 and FaDu cells. Red fluorescence showed that the cells were in the proliferative phase. The ratio of red cells to total cells in Edu staining indicates the ratio of cells in the proliferative phase. *n* = 3. Scale bars, 100 μm. (**E**,**F**) Cell cycle flow cytometry results (**E**) and quantitative analysis (**F**) in NC and DRC groups of CAL27 and FaDu cells. *n* = 3. * *p* < 0.05, ** *p* < 0.01, and *** *p* < 0.001.

Despite surgical or drug treatments, cancer often relapses and metastasizes. The temporary halt in tumor progression during this period can be explained by cancer dormancy [31]. When external conditions are unfavorable for growth, a small subset of cancer cells can enter a reversible, slow-proliferation state to evade chemotherapy [32]. We observed a simultaneous decrease in glucose consumption and lactate production in DRCs (Figure 2A). The results suggest a significant reduction in the basic metabolic level of DRCs, exhibiting a state akin to “quiescence”. To support this point, we conducted Western blot experiments on cell cycle proteins to assess their levels. Cell proliferation relies on the progression through the four phases of G0/G1, S, G2, and M. DDP exerts its anti-tumor effects by interfering with DNA replication and causing DNA damage. Consequently, could these quiescent cells evade the therapeutic effects of chemotherapy by avoiding entry into the S phase of DNA replication? Cyclin E1 can facilitate the phosphorylation of retinoblastoma protein (pRB) and initiate cell replication and G1/S phase transition by binding with complexes of cyclins and cyclin-dependent kinase-2 (CDK-2). However, this phosphorylation is also subject to inhibition by p21 [33]. Subsequently, we observed a decrease in the phosphorylation of cell cycle-promoting proteins (pRB and CyclinE1) and an increase in the content of the cell cycle-inhibiting protein p21 in DRCs (Figure 2B). The proportion of cells in the proliferative phase was reduced, as shown by EdU detection (Figure 2C). EdU is a thymidine analog that, during cellular DNA replication, can enter the cell nucleus and incorporate with DNA, forming the EdU-DNA complex. The intensity of this complex can be measured to determine the level of cellular proliferation activity. These results indicated that DRCs were indeed in a state of slow-proliferation dormancy. Furthermore, flow cytometry analysis of cell cycle phases revealed a significant increase in the proportion of cells in the G0/G1 phase in DRCs, with a large number of cells not entering the DNA replication peak in the S phase (Figure 2E,F). Mitophagy guides cells into a dormant state characterized by slow DNA replication and proliferation, helping cells reduce material exchange with the external environment and self-consumption, thereby evading the adverse effects of chemotherapy drugs.

### 3.3. Tumor Dormancy Terminates after DDP Treatment

**Figure 3:**
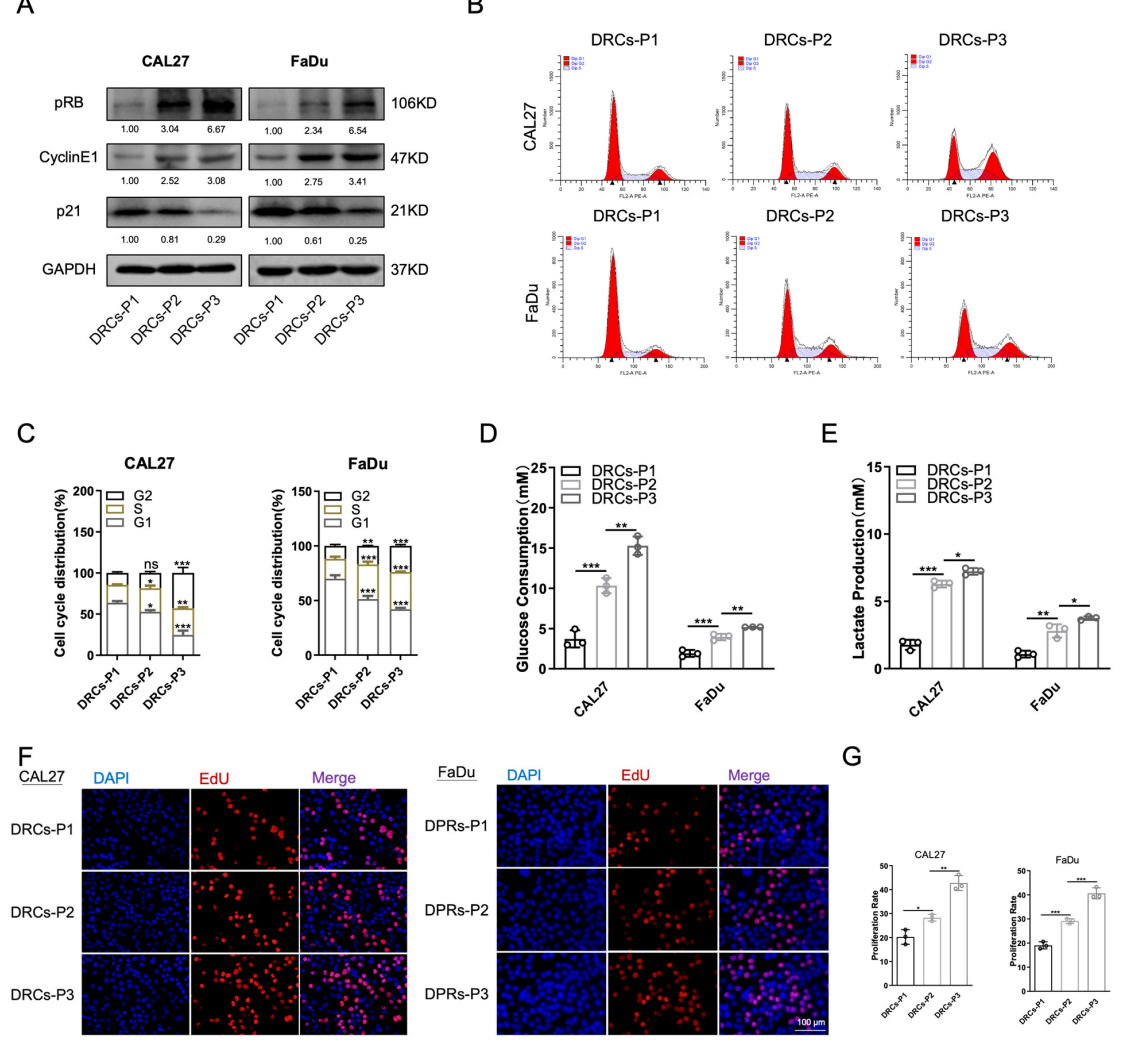
(**A**) Western blot results for cell cycle proteins in generations P1, P2, and P3 of DRCs of CAL27 and FaDu cells. (**B**,**C**) Cell cycle analysis flow cytometry results (**B**) and quantitative analysis (**C**) for generations P1, P2, and P3 of DRCs of CAL27 and FaDu cells. *n* = 3. (**D**,**E**) Glucose consumption and lactate production in generations P1, P2, and P3 of DRCs of CAL27 and FaDu cells. *n* = 3. (**F, G)** Representative images (**F**) and quantitative analysis (**G**) of Edu experiments in generations P1, P2, and P3 of DRCs of CAL27 and FaDu cells. *n* = 3. Scale bars, 100 μm. * *p* < 0.05, ** *p* < 0.01, and *** *p* < 0.001.

The recurrence and distant metastasis of tumors after chemotherapy are major contributors to the failure of cancer treatment. These are currently the leading causes of cancer death [34]. Recurrent cancer cells exhibit heightened adaptability and treatment resistance. It was hypothesized that tumor cells enter a dormant state through mitochondrial autophagy to evade DDP cytotoxicity. After the cessation of chemotherapy, these cells terminate their slow-proliferation status and undergo a resurgence. To test this hypothesis, we stopped stimulating DRCs with DDP and proceeded with regular subculturing. Analysis of cell cycle-related proteins showed a gradual increase in Cyclin E1 levels, accompanied by a gradual decrease in p21 content. This resulted in an elevated level of pRB, indicating a progressively accelerated cycle in DRCs following chemotherapy (Figure 3A). Next, we conducted flow cytometry analysis. The results indicate a decrease in the proportion of cells in the G0/G1 phase and a gradual increase in the proportion of cells actively replicating DNA in the S phase within the DRC population (Figure 3B,C). The Edu results further support this, showing a gradual increase in the proportion of cells in the actively proliferating phase (Figure 3F,G). As the cell cycle accelerated, cells transitioned from a state of slow proliferation. Simultaneously, we investigated whether the baseline metabolic levels of the cells had recovered or increased. Energy intake and lactate production were assessed across generations following the cessation of DDP treatment. The elevated glucose consumption and lactate production in DRCs indicate a gradual recovery of metabolic levels in the absence of DDP (Figure 3D,E). These findings suggest that DRCs, after the cessation of chemotherapy, exit the dormant state, with proliferative activity and metabolic levels progressively increasing over subsequent generations.

### 3.4. Resurgent DRCs Exhibit a Heightened Malignant Phenotype

**Figure 4:**
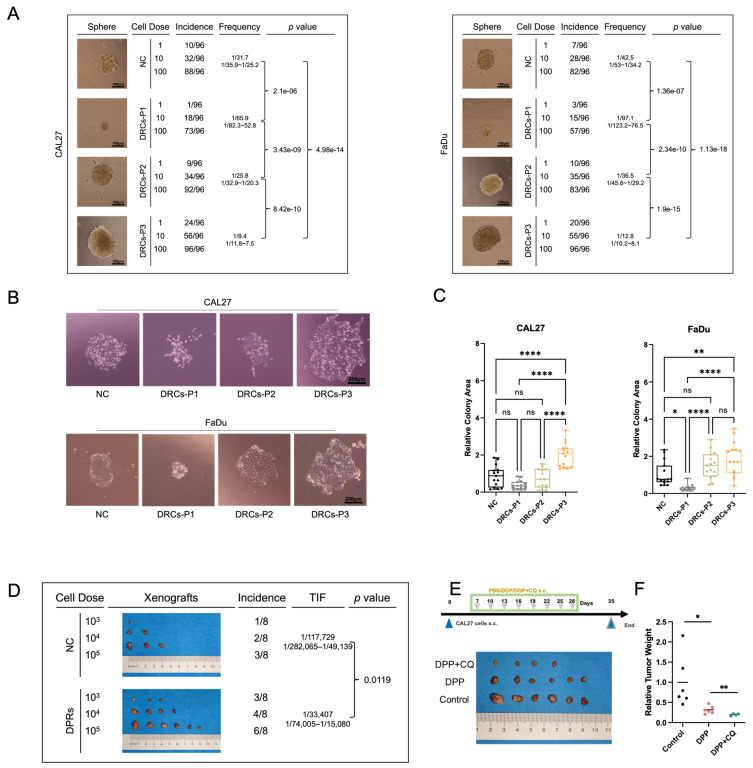
(**A**) Representative images and analysis of limiting dilution assay of sphere formation of CAL27 and FaDu cells. (**B**,**C**) Representative images (**B**) and relative colony area (**C**) of colony formation of CAL27 and FaDu cells. (**D**) Results of limiting dilution assay of tumorigenesis in vivo (CAL27). *n* = 8. (**E**,**F**) Results (**E**) and relative tumor weight (**F**) of mouse xenografts in NC, DDP, and DDP + CQ groups (CAL27). *n* = 6. ns, not significant. * *p* < 0.05, ** *p* < 0.01, and **** *p* < 0.0001.

Mitophagy facilitates the entry of HNSCC cells into a dormant state, inducing the formation of DRCs. These DRCs exhibit the termination of dormancy and a gradually accelerated cell cycle after DDP treatment. To further explore the CSC properties of dormant DRCs, we conducted a limiting dilution sphere-forming assay. In consecutive passages following the cessation of dormancy, DRCs acquired progressively enhanced limiting dilution sphere-forming capacity (Figure 4A), possibly linked to the gradual termination of dormancy. Notably, at P3, they exhibited a significantly stronger sphere-forming ability than the NC group, suggesting that DRCs possess stronger stem cell characteristics when returning to normal physiological states. Next, we investigated the tumor-initiating potential of DRCs using a colony formation assay, and we found that, post-recovery, DRCs displayed a heightened colony formation ability (Figure 4B,C). The limiting dilution assay in vivo can verify the tumorigenic ability of progressively diluted cancer cells. This is an experiment that reflects the CSC properties of cells. In vivo experiments also demonstrated the increased CSC properties and tumor-forming capacity of DRCs (Figure 4D). In conclusion, mitochondrial autophagy-induced DRCs, gradually recovering in the absence of DDP, exhibit a heightened malignant profile. In addition, we utilized the autophagy inhibitor chloroquine to suppress autophagy during DDP treatment. The synergistic effect of autophagy inhibition and chemotherapy achieved a superior anti-tumor outcome (Figure 4E,F).

## 4. Discussion

**Figure 5:**
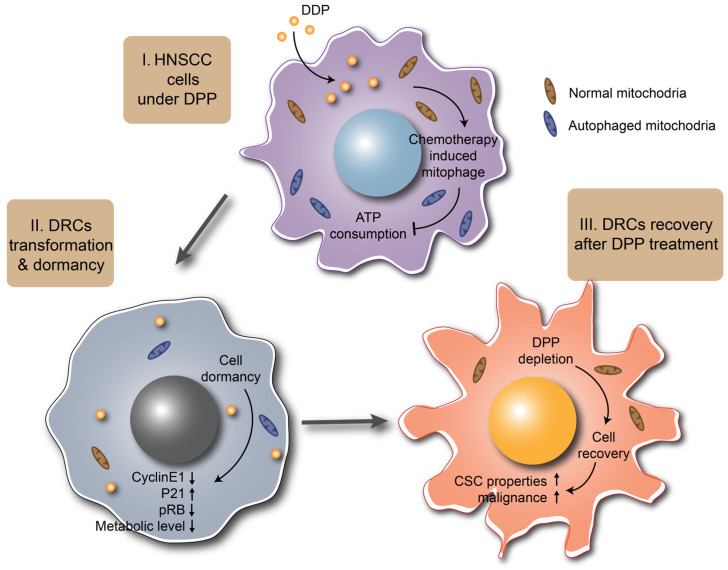
Schematic diagram of dormancy and recovery of mitophagy-induced DRCs in HNSCC. During DDP (Cisplatin) treatment, mitophagy induces head and neck squamous cell carcinoma (HNSCC) cells to enter a dormant state characterized by low metabolism and reduced proliferation. Following the cessation of DDP induction, dormant cells transition back to a proliferative state and exhibit heightened malignancy and cancer stem cell (CSC) characteristics.

Post-treatment metastasis of cancer is the most lethal aspect of cancer [4]. It involves the dormancy and colonization of tumor cells [35]. The high heterogeneity and metabolic reprogramming within cancer cells give them the ability to evade cancer therapies. Dormant cells are essential in this process. They settle at distant sites and do not form detectable macroscopic lesions until months or even years after relapse. During this time, dormant cells maintain their state through epigenetic regulation, such as chromatin modifications and enhanced DNA methylation. This leads to a slowdown or arrest in the cell cycle, enhanced cellular stress responses, and activation of autophagy [36]. Simultaneously, dormant cells can re-enter the cell cycle, exhibiting drug resistance and heightened malignancy. Through dormancy, cancer cells gain increased survival capabilities to resist adverse external factors [37,38]. In our study, we demonstrated that mitophagy is associated with the transition of HNSCC into a low-metabolism, low-proliferation dormant state during DDP treatment, endowing the tumor with enhanced drug resistance and CSC characteristics. Meanwhile, the recovery of tumor dormancy results in increased tumorigenicity (Figure 5).

Mitophagy, a process essential for maintaining cellular homeostasis, has been identified as a significant contributor to the development of DDP resistance in dormant cells in our research. This resistance, in turn, contributes to the acquisition of CSC properties and the emergence of enhanced malignancy during the subsequent recovery phase. To address this phenomenon and improve the efficacy of DDP treatment, we have investigated the concurrent use of autophagy inhibitors. The use of autophagy inhibitors in combination with DDP therapy has shown promise in targeting and eliminating dormant tumor cells. This combination not only significantly amplifies the cytotoxic effects of cisplatin but also proves to be particularly effective in eradicating cells that have entered a quiescent state. The observed outcome is a substantial enhancement in the overall efficacy of cisplatin, leading to a noteworthy regression and disappearance of subcutaneous tumors. This approach emphasizes the significance of comprehending the complex relationship between mitochondrial autophagy and drug resistance. It also offers a potential therapeutic solution for addressing the obstacles presented by dormant tumor cells. The disappearance of subcutaneous tumors highlights the clinical relevance and potential translational impact of co-administering autophagy inhibitors with cisplatin, providing new possibilities for more effective cancer treatment strategies.

Recent research indicates the complex role of mitophagy in cancer. Deng et al. proposed that the depletion of mitophagy leads to the accumulation of damaged mitochondria and increased ROS under hypoxic conditions, which results in the activation of inflammasomes, abnormal secretion of soluble cytokines, and impaired osteoclast maturation, and ultimately promotes bone metastasis in breast cancer [39]. Rademaker et al. suggested that targeting Myoferlin induces an increase in mitochondrial autophagy and the accumulation of ROS, triggering lipid peroxidation and cell ferroptosis [40]. Meng et al. uncovered that the loss of the E3 ubiquitin ligase CRL4 in ovarian cancer leads to slowed proliferation and the loss of chemotherapy resistance capabilities by upregulating mitophagy through the Parkin-PINK1 pathway [41]. These results suggest that mitophagy may be detrimental to tumors. However, Alcalá et al. uncovered that mitophagy and metabolic impairment disrupt the metabolic plasticity of pancreatic CSCs, increasing sensitivity to metformin [42]. Li et al. proposed that PINK1-mediated mitophagy promotes redox homeostasis, maintaining drug resistance in lung adenocarcinoma [43]. Yao et al. discovered that the inhibition of CDK9 leads to the suppression of the SIRT1-FOXO3-BNIP3 axis, resulting in the inhibition of PINK1-PRKN-mediated mitophagy, which helped overcome drug resistance in hepatocellular carcinoma [44]. Mitophagy appears to play a dual role in tumors. Uncontrolled mitophagy can disrupt the stability of tumor cells, increasing tumor cell death. On the other hand, mitophagy provides tumor cells with metabolic plasticity, enhancing adaptability to external pressure and chemotherapy. In our study, mitophagy is crucial for DDP-mediated chemotherapy resistance and induces HNSCC cells to enter a dormant state.

Cancer cell dormancy is a process in which the cell cycle enters a reversible slowing state or comes to a complete halt. The behavior of DRCs resembles that of a subset of CSCs, specifically a dormant subgroup that engages in minimal energy metabolism to sustain life. These cells exhibit slowed or halted proliferation to evade external environmental stimuli and can transition between dormant and proliferative states [23]. Dormancy in cancer is critical for the accumulation of genetic mutations, evasion of immune surveillance, resistance to tumor treatments, and survival and metastasis at distant sites [45]. Dormant cancer cells are considered a cause of both primary tumor progression and metastatic recurrence. There are currently two potential directions for advancing research on dormant cells. The first is to maintain the dormant state of cancer cells without progression, while the second involves activating dormant cells to enhance their sensitivity during cancer treatment. Clearing inactive dormant cells in cancer to address tumor metastasis has been a longstanding challenge in the scientific community [36]. Compared to rapidly proliferating tumor cells, dormant cells have weakened interactions with the external environment, resulting in lower responsiveness to chemotherapy, radiotherapy, and emerging immunotherapies [46,47,48]. Drug development for dormant cells is also underway [49]. The mechanisms underlying dormant cell formation lack a clear definition, and research in this area is ongoing. Here, we propose the significant role of mitophagy in the dormancy and chemotherapy resistance of HNSCC. Combining autophagy inhibitors with chemotherapy to reduce the formation of dormant cells and decrease the metabolic plasticity of tumor cells offers a new avenue for future research.

## Data Availability

Data will be made available on request.
